# Association between pharmacological guideline adherence and actigraphy‐measured sleep variables in long‐term hospitalized patients with schizophrenia

**DOI:** 10.1002/pcn5.70154

**Published:** 2025-07-10

**Authors:** Kentaro Saito, Yusuke Arai, Daimei Sasayama, Toshinori Nakamura, Kazuhiro Suzuki, Mika Koido, Reiko Sahara, Yuka Nakajima, Aya Horiuchi, Fumiya Fukui, Kazuaki Kuraishi, Shinsuke Washizuka

**Affiliations:** ^1^ Department of Psychiatry Kurita Hospital Nagano Japan; ^2^ Department of Psychiatry Shinshu University School of Medicine Matsumoto Japan; ^3^ Department of Community Mental Health Shinshu University School of Medicine Matsumoto Japan

**Keywords:** guideline, hypnotic, pharmacotherapy, sleep, schizophrenia

## Abstract

**Aim:**

Improving sleep quality is a crucial clinical objective in schizophrenia care; however, the association between evidence‐based pharmacological treatment and sleep outcomes remains unclear. This study aimed to examine whether guideline adherence, assessed using the individual fitness score (IFS), correlated with actigraphy‐measured sleep in long‐term hospitalized patients.

**Methods:**

We included 40 inpatients aged <65 years who were diagnosed with schizophrenia. Guideline adherence was assessed using the IFS and actigraphy was used to measure total sleep time (TST), sleep latency (SL), and sleep efficiency (SE). In 33 patients, these measures were re‐evaluated after 6 months.

**Results:**

Cross‐sectional analysis showed a positive correlation between IFS and TST (rho = 0.362, *P* = 0.022), which persisted after adjusting for Brief Psychiatric Rating Scale scores (*β* = 0.318, *P* = 0.024). IFS was not associated with SL or SE, and differences in hypnotic use did not significantly affect the sleep parameters. Additionally, longitudinal changes in IFS over 6 months were not significantly associated with changes in TST, SL, or SE (all *P* > 0.05).

**Conclusion:**

In this cohort of long‐term hospitalized patients with schizophrenia, guideline adherence (higher IFS) was associated with increased TST. However, no clear longitudinal effects of IFS on sleep were observed. These findings emphasize the importance of guideline adherence in optimizing sleep, although extended follow‐ups and larger sample sizes are required to confirm the long‐term impact of guideline‐based treatments on sleep in patients with schizophrenia.

## Graphical abstract

Correlation between individual fitness score (IFS) and total sleep time (TST). A significant positive correlation was observed between TST and IFS (rho = 0.362, P = 0.022).

## INTRODUCTION

Schizophrenia is a chronic mental disorder characterized by positive symptoms, such as hallucinations and delusions, and negative symptoms, including flat affect and social withdrawal, which significantly impair patients' quality of life.^1^ Among the challenges faced by individuals with schizophrenia, sleep disturbances are particularly prevalent and widely reported.^2^, ^3^, ^4^, ^5^ These disturbances include insomnia, fragmented sleep, reduced total sleep time (TST), decreased sleep efficiency (SE), and prolonged sleep latency (SL).^6^, ^7^, ^8^ Such changes not only exacerbate daytime cognitive dysfunction and emotional dysregulation but also worsen schizophrenia symptoms and hinder treatment outcomes. Therefore, addressing sleep disturbance is a critical objective in the comprehensive care of patients with schizophrenia.^9^

Antipsychotic medications are known to have beneficial effects on sleep quality. Certain antipsychotics have been shown to improve sleep architecture by reducing SL, increasing TST, and enhancing SE.^10^, ^11^, ^12^ However, the use of benzodiazepines and hypnotics for the treatment of insomnia in patients with schizophrenia remains controversial. Although these medications may provide short‐term symptom relief, their long‐term use is associated with risks such as tolerance, dependence, and adverse effects on treatment outcomes.^13^, ^14^ The efficacy and safety of the recently introduced orexin receptor antagonists and melatonin receptor agonists on sleep parameters in patients with schizophrenia are yet to be elucidated. Under these circumstances, the schizophrenia pharmacological treatment guidelines in Japan explicitly discourage the routine use of hypnotics, advocating instead for monotherapy with appropriately dosed antipsychotics.^15^ Nevertheless, hypnotics continue to be frequently prescribed in clinical practice, reflecting the gap between the guideline recommendations and real‐world adherence.^16^

To address these challenges, there is the Effectiveness of GUIdeline for Dissemination and Education in psychiatric treatment (EGUIDE) project. This project is a nationwide, 1‐day intensive training program designed to educate psychiatrists on the treatment guidelines for schizophrenia and major depressive disorder. Previous studies have shown that the program not only improves participants' understanding of evidence‐based treatments,^17^ but also leads to sustained changes in clinical practice: guideline‐adherent behaviors increase 1 year after training and remain elevated 2 years later.^18^ Among patients treated by trained psychiatrists, prescribing trends have shifted over time, with increased antipsychotic monotherapy and reduced use of anxiolytics and hypnotics for schizophrenia, as well as increased antidepressant monotherapy for depression.^19^ These findings suggest that the EGUIDE program promotes better prescribing practices and supports broader adoption of guideline‐based psychiatric care. The EGUIDE project introduced the individual fitness score (IFS),^20^ a novel metric designed to quantify adherence to schizophrenia treatment guidelines. The IFS is not only a clinical tool but also a modifiable factor that provides actionable feedback to clinicians to enhance evidence‐based treatment practices. Previous research has established the IFS as a robust indicator of treatment quality, with higher scores correlating with better clinical outcomes. For instance, patients with higher IFS exhibit lower Positive and Negative Syndrome Scale scores, reflecting reduced symptom severity.^21^ Additionally, IFS is associated with better social functioning, such as increased working hours.^22^ These findings underscore the potential of the IFS as a tool for bridging the gap between guideline recommendations and clinical practice.

Building on these insights, this study focuses on sleep as a critical clinical outcome. Previous studies have examined the relationships between schizophrenia, sleep disorders, and pharmacological interventions. However, the direct connections between the use of hypnotic agents in real‐world clinical practice, adherence to treatment guidelines (assessed by the IFS), and sleep quality remain insufficiently understood. We therefore hypothesized that, among patients with schizophrenia, adherence to pharmacological treatment guidelines, rather than the use of hypnotic agents, may have a greater impact on objective sleep quality. Specifically, lower IFS scores (i.e., lower adherence to treatment guidelines) can lead to increased drug interactions and daytime drowsiness, thereby adversely affecting nighttime sleep quality. Conversely, higher IFS scores (reflecting closer adherence to antipsychotic monotherapy) may reduce these risks and enhance sleep quality. This hypothesis posits that higher IFS scores, which indicate optimal antipsychotic use and reduced hypnotic reliance, are associated with improved sleep quality.

To test this hypothesis, we examined the relationship between the IFS and sleep variables measured objectively using actigraphy, including TST, SL, and SE. This study aimed to provide evidence supporting the role of guideline‐compliant treatments in improving sleep quality in patients with schizophrenia.

## METHODS

### Study design and patients

This single‐center prospective observational study aimed to evaluate the association between the IFS and sleep variables measured using actigraphy in patients with chronic schizophrenia undergoing long‐term hospitalization. The study was conducted at a non‐public psychiatric hospital in Japan and involved patients aged <65 years, all diagnosed with schizophrenia by a treating psychiatrist. Based on medical records, the treating psychiatrist confirmed the diagnosis of schizophrenia according to the Diagnostic and Statistical Manual of Mental Disorders, Fifth Edition criteria.^23^ Participants were limited to those who had been continuously hospitalized for over 1 year, reflecting the unique medical environment of long‐term hospitalization in Japan.^24^ Forty patients were enrolled, and the IFS was calculated for all participants at the inclusion timepoint (T1: February to April 2024). The sleep variables were assessed using actigraphy at this time point. Among the 33 patients who could be followed up, the IFS was recalculated 6 months later (T2: August to October 2024), along with a repeated assessment of sleep variables using actigraphy. This study was approved by the Ethics Committee of Kurita Hospital and conducted in accordance with the Declaration of Helsinki. All participants received a detailed explanation of the purpose, methodology, potential benefits and risks, and data protection measures of the study. Subsequently, written informed consent was obtained. Only the patients whose participation was deemed safe by their attending psychiatrists were included in this study.

### Procedures and measures

At T1, the demographic and clinical data of the patients were collected. Specifically, patient background information such as age, sex, age at onset, years of education, medical history, and treatment details (including the types and usage of antipsychotics, hypnotics, mood stabilizers, and anticholinergics) were obtained from electronic medical records. The current IFS was calculated based on the prescription content, and treatment‐resistant schizophrenia (TRS) was retrospectively identified through a review of medical records. In Japan, TRS is defined as a condition in which a patient has “never achieved a Global Assessment of Functioning score of 41 or higher” despite having received “at least two antipsychotic medications” at a dosage equivalent to “more than 600 mg/day of chlorpromazine” for “more than four weeks.”^25^ The IFS was scored based on the criteria of Inada et al.^20^ as follows: The IFS ranged from 0 to 100, with higher scores indicating better adherence to the recommended practices. The evaluation criteria differed for TRS and non‐TRS. For patients without TRS, monotherapy with second‐generation antipsychotics scored 100 points, whereas for patients with TRS, clozapine monotherapy was required to achieve a perfect score. The absence of clozapine use in patients with TRS incurred a 60‐point penalty. Furthermore, the use of one hypnotic resulted in a 15‐point reduction, while the use of two hypnotics led to a 35‐point reduction. If the cumulative score fell below 0, it was capped at 0. This deduction‐based system evaluated guideline adherence with increased hypnotic use, resulting in lower scores.

At both T1 and T2, we assessed the IFS, Brief Psychiatric Rating Scale (BPRS),^26^ and sleep variables measured using actigraphy. Sleep data were collected continuously over 1 week. During the actigraphy evaluation, the use of rescue medications was generally prohibited, and patients were instructed to wear the actigraphy device nightly. The recorded parameters included SL, TST, and SE. The TST was calculated as the total duration of epochs scored as sleep between bedtime and wake‐up time. SE was determined as the ratio of TST to the total time spent in bed (i.e., the time between bedtime and wake‐up time) and expressed as a percentage. Bedtime and wake‐up times were determined by experienced technicians, who validated their accuracy using self‐reported sleep diaries and corresponding data on activity levels and light intensity. Sleep and wake states were analyzed using the Cole–Kripke algorithm in ActiLife 6 software (ActiGraph).

### Statistical analysis

Statistical analyses were performed using SPSS version 29. Statistical significance was set at a two‐tailed P < 0.05.

This study was exploratory, as no prior research has evaluated the association between IFS and sleep variables. For the cross‐sectional analysis, power analysis using G*Power 3.1 determined a target sample size of 34, based on an effect size of rho = 0.45 (Fisher's *Z* transformation), statistical power of 1 − *β* = 0.80, and significance level at *α* = 0.05. The longitudinal analysis was designed to generate hypotheses for future large‐scale studies; no separate sample size calculations were conducted.

A basic statistical table summarizing the demographic and clinical characteristics of patients with schizophrenia at T1 (*N* = 40) and T2 (*N* = 33) was created using descriptive statistics (Table 1). The Shapiro–Wilk test indicated that most variables were not normally distributed; therefore, all variables were described using medians and interquartile ranges (IQR) or percentages. At T1, we first classified all patients with schizophrenia (*N* = 40) into three groups: those not using hypnotics, those using one type of hypnotic, and those using two or more types of hypnotics. To examine the differences in sleep variables (SL, TST, and SE) between the groups, we conducted a Kruskal–Wallis test. Spearman's rank correlation was used to examine the association between the IFS and sleep variables in all patients with schizophrenia. Additionally, given that previous studies demonstrated a relationship between the IFS and schizophrenia symptoms,^21^ linear regression analyses were conducted to examine the impact of the IFS on each sleep variable, adjusting for BPRS as a confounder.

**Table 1:** Demographic and clinical characteristics at each time point.

Characteristic	All participants at T1 (*N* = 40)	Participants followed up until T2 (*N* = 33)
**Background**	**Median (IQR) or** ***N*** **(%)**	
Age (years)	55.5 (49.5–61.8)	56.0 (51.0–61.0)
Sex (male), *N* (%)	24 (60.0%)	22 (66.7%)
Body mass index (kg/m^2^)	22.3 (18.3–23.7)	21.3 (18.3–23.5)
Age at onset (years)	20.0 (18.8–25.5)	20.0 (18.3–24.5)
Years of education (years)	12.0 (12.0–14.0)	12.0 (12.0–14.0)
Duration of hospitalization (years)	7.3 (4.0‐11.3)	7.7 (4.1–12.0)
Treatment‐resistant schizophrenia, *N* (%)	8 (20.0%)	8 (24.2%)
**Pharmacotherapy**	**Median (IQR) or** ***N*** **(%)**	
Antipsychotic, *N* (%)/CP‐eq (mg)	40 (100%)/955.3 (604.5–1368.9)	33 (100%)/900.0 (600.0–1506.8)
Clozapine, *N* (%)	0 (0%)	2 (6.1%)
Mood stabilizer, *N* (%)	15 (37.5%)	13 (39.4%)
Benzodiazepine, *N* (%)	17 (42.5%)	14 (42.4%)
Anticholinergic, *N* (%)	14 (35.0%)	8 (24.2%)
Orexin receptor antagonist, *N* (%)	15 (37.5%)	13 (39.4%)
Melatonin receptor agonist, *N* (%)	6 (15.0%)	4 (12.1%)
Number of classes of hypnotic medications		
0, *N* (%)	12 (30.0%)	12 (36.4%)
1, *N* (%)	20 (50.0%)	13 (39.4%)
≥2, *N* (%)	8 (20.0%)	8 (24.2%)
IFS	35.0 (0–65.0)	40.0 (0–85.0)
**Symptom rating scales**	**Median (IQR)**	
BPRS	15.0 (5.3–23.0)	9.0 (4.5–13.0)
**Sleep variables based on actigraphy**	**Median (IQR)**	
TIB (min)	523.4 (439.1–541.6)	479.0 (451.5–525.8)
TST (min)	428.8 (364.8–497.2)	415.7 (365.0–467.9)
SE = TST/TIB (%)	85.5 (78.4–92.5)	87.6 (79.7–92.2)
SL (min)	2.1 (0.1–6.7)	0.8 (0–4.6)

Abbreviations: BPRS, Brief Psychiatric Rating Scale; CP‐eq, chlorpromazine‐equivalent; IFS, individual fitness score; IQR, interquartile range; TIB, time in bed; TST, total sleep time; SE, sleep efficiency; SL, sleep latency.

Longitudinal associations were analyzed using a linear regression model for the subset of patients who could be followed up (*N* = 33). Changes in sleep variables (ΔSL, ΔTST, and ΔSE) were used as dependent variables, where Δ represents the difference between T2 and T1 values. Changes in IFS (ΔIFS) were used as the explanatory variable and changes in BPRS (ΔBPRS) as a covariate. This analysis explored the potential impact of changes in treatment adherence (IFS) and psychiatric symptom severity (BPRS) on longitudinal sleep variables. Although age was initially considered as a potential explanatory variable, an exploratory analysis revealed no significant correlation between age and IFS at baseline (rho = 0.097, *P* = 0.553), therefore we did not include age in the final linear model.

Given the small sample size, the bootstrap method was used for all linear regression analyses, including longitudinal analyses, to estimate confidence intervals. At T2, four patients were discharged from the hospital and could not be followed. The remaining three patients could not participate in the follow‐up assessment due to worsening psychiatric symptoms—one due to catatonia and two due to agitation. Baseline characteristics (age, IFS, BPRS, and sleep variables) at T1 were compared between the seven participants who dropped out at T2 and the 33 who continued, using the Mann–Whitney *U* test. No significant differences were observed between groups (Table S1), therefore 33 participants were considered representative of the population and missing data were assumed to be missing at random. A comparison of sleep parameters, medication, and BPRS scores between T1 and T2 for the 33 patients who completed follow‐up was conducted (Table S2). Fisher's exact test was used for categorical variables and the Wilcoxon signed‐rank test for continuous variables. While there were no significant changes in medication, BPRS scores and SL showed statistically significant improvements. Specifically, the BPRS scores decreased from a median of 18.0 (IQR: 6.0–23.5) at T1 to 9.0 (IQR: 4.5–13.0) at T2 (*P* = 0.002), and SL improved from 1.6 min (IQR: 0.2–6.9) at T1 to 0.8 min (IQR: 0–4.6) at T2 (*P* = 0.042). The bootstrap procedure was repeated 1000 times. This number was selected to ensure the precision of the confidence interval estimation while maintaining the computational load within a reasonable range. This setting is widely used in many statistical analyses and was deemed appropriate for this study.

## RESULTS

### Demographic and clinical characteristics

**Figure 1:**
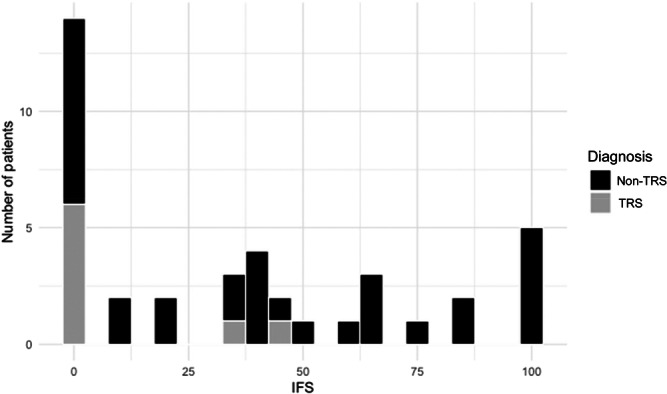
Distribution of IFS scores at T1 in patients with schizophrenia. The median IFS was 35.0 (interquartile range: 0–65.0). IFS, individual fitness score; TRS, treatment‐resistant schizophrenia.

Table 1 shows the baseline demographic and clinical characteristics at T1 for all participants (*N* = 40) and for those followed up until T2 (*N* = 33). The median age of participants was 55.5 years (IQR: 49.5–61.8), with males comprising 60.0% (24/40) of the cohort. The median body mass index (BMI) was 22.3 (IQR: 18.3–23.7) and the median age at the onset of schizophrenia was 20.0 years (IQR: 18.8–25.5). The participants had a median of 12.0 years of education (IQR: 12.0–14.0). The median duration of hospitalization among participants was 7.3 years (IQR: 4.0–11.3), reflecting the context of long‐term psychiatric hospitalization in Japan. TRS was observed in 20.0% (8/40) of participants. All participants were prescribed antipsychotics, with a median chlorpromazine‐equivalent (CP‐eq)^27^ dose of 955.3 mg/day (IQR: 604.5–1368.9). None of the participants were prescribed clozapine. Mood stabilizers were used by 37.5% (15/40) and benzodiazepines were used by 42.5% (17/40). Anticholinergics were prescribed to 35.0% (14/40) of patients, orexin receptor antagonists to 37.5% (15/40), and melatonin receptor agonists to 15.0% (6/40). Hypnotic medications are summarized according to three pharmacological classes of action (benzodiazepines and *Z*‐drugs, orexin receptor antagonists, and melatonin receptor agonists). Twelve patients (30.0%) used no hypnotic medication, 20 (50.0%) used one hypnotic medication, and six patients (20.0%) used two or more hypnotic medications. The median IFS was 35.0 (IQR: 0–65.0). Figure 1 shows the distribution of the IFS scores. Symptom severity, measured using the BPRS, showed a median score of 15.0 (IQR: 5.3–23.0). Sleep parameters assessed using actigraphy revealed the following values. The median time in bed (TIB) was 523.4 min (IQR: 439.1–541.6) and the median TST was 428.8 min (IQR: 364.8–497.2). SE had a median value of 85.5% (IQR: 78.4–92.5) and SL was notably low, with a median of 2.1 min (IQR: 0.1–6.7).

At T2, the median age of participants was 56.0 years (IQR: 51.0–61.0), with males comprising 66.7% (22/33). The median BMI was 21.3 (IQR: 18.3–23.5) and the median age at the onset of schizophrenia was 20.0 years (IQR: 18.3–24.5). Additionally, the median years of education was 12.0 (IQR: 12.0–14.0). TRS accounted for 24.2% (8/33) of the patients. Moreover, 100.0% of participants were prescribed antipsychotics, with a median CP‐eq dose of 900.0 mg/day (IQR: 600.0–1506.8). Clozapine was prescribed to 6.1% (2/33) of patients. Mood stabilizers were used by 39.4% (13/33) of patients and benzodiazepines were used by 42.4% (14/33). Anticholinergics were prescribed to 24.2% (8/33) of patients, orexin receptor antagonists to 39.4% (13/33), and melatonin receptor agonists to 12.1% (4/33). At T2, 12 patients (36.4%) used no hypnotic medication, 13 (39.4%) used one hypnotic medication, and eight patients (24.2%) used two or more hypnotic medications. The median IFS at T2 was 40.0 (IQR: 0–85.0). At T2, the median TIB was 479.0 min (IQR: 451.5–525.8), the median TST was 415.7 min (IQR: 365.0–467.9), the median SE was 87.6% (IQR: 79.7–92.2), and the median SL was 0.8 min (IQR: 0–4.6).

### Association between the number of hypnotic classes used and sleep variables (SL, TST, and SE) in patients with schizophrenia

**Figure 2:**
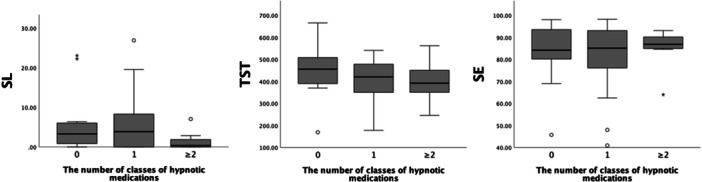
Box‐and‐whisker plots comparing sleep onset latency (SL), total sleep time (TST), and sleep efficiency (SE) among patients categorized into three groups based on the number of hypnotic agents used (0, 1, or ≥2). No significant differences in SL, TST, or SE were observed among these groups.

We used the Kruskal–Wallis test to compare the sleep variables in the three groups classified based on the number of hypnotic agents used (Figure 2). No significant differences in SL, TST, or SE were observed between these groups (i.e., no use of hypnotics vs. one agent vs. two or more agents; Tables S3–S5).

### Associations between IFS and sleep variables (SL, TST, and SE) in patients with schizophrenia

**Figure 3:**
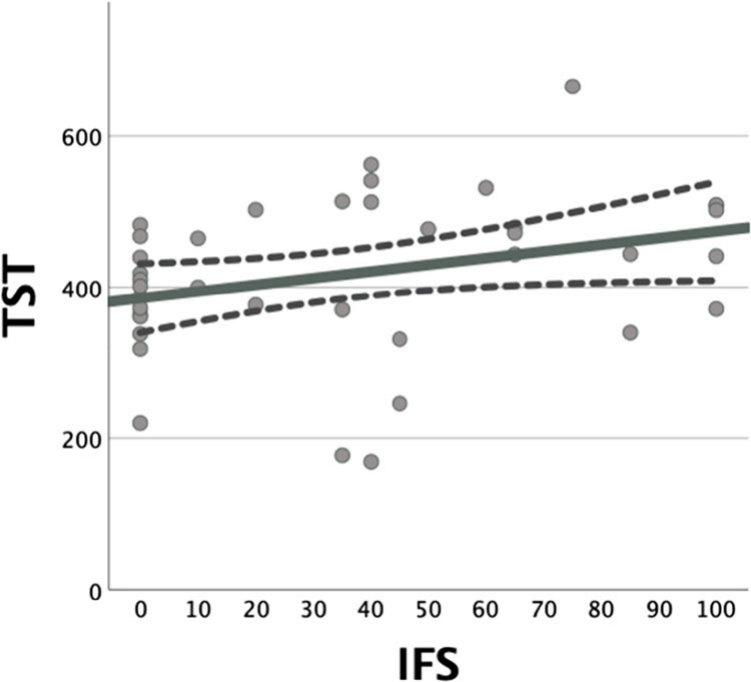
Correlation between IFS and TST. A significant positive correlation was observed between TST and IFS (rho = 0.362, P = 0.022). IFS, individual fitness score; TST, total sleep time.

We investigated the correlations between the IFS and sleep variables in all patients with schizophrenia (*N* = 40). A significant positive correlation was observed between the IFS and TST (rho = 0.362, *P* = 0.022) (Figure 3). Conversely, no significant correlations were observed for SL (rho = −0.053, *P* = 0.746) or SE (rho = 0.236, *P* = 0.143) (Table 2). Subsequently, we analyzed each sleep variable as a dependent variable using a linear regression model adjusted for the BPRS to control for confounding effects. The analysis revealed a significant association between IFS and TST (*β* = 0.318, *P* = 0.024). However, no significant associations were found for SL (*β* = −0.187, *P* = 0.290) or SE (*β* = 0.175, *P* = 0.207) (Table 3). In addition, to address potential bias from dropouts, we repeated these analyses in the completer‐only sample (*n* = 33); the corresponding results for Tables 2 and 3 are provided in Tables S6 and S7).

**Table 2:** Correlation between IFS and sleep variables.

Variable	Rho	P‐value	95% CI (lower–upper)
**IFS‐TST**	**0.362**	**0.022** *	**0.047–0.611**
IFS‐SL	−0.053	0.746	−0.367–0.272
IFS‐SE	0.236	0.143	−0.091–0.517

Abbreviations: IFS, individual fitness score; SE, sleep efficiency; SL, sleep latency, TST, total sleep time.

* Two‐tailed P < 0.05.

**Table 3:** Linear regression analysis adjusting for BPRS: association between IFS and sleep variables.

Independent variable	*B*	*β*	*P*‐value	95% CI for *B* (lower–upper)
**Dependent variable: SL**				
(Constant)	10.673		0.052	3.142–19.233
IFS	−0.049	−0.187	0.290	−0.144–0.025
BPRS	−0.210	−0.218	0.179	−0.505–0.033
**Dependent variable: TST**				
Constant	379.789		<0.001	308.826–458.172
**IFS**	**0.916**	**0.318**	**0.024** *	**0.163–1.624**
BPRS	0.312	0.029	0.866	−3.617–3.641
**Dependent variable: SE**				
Constant	78.909		<0.001	68.861–88.592
IFS	0.068	0.175	0.207	−0.048–0.177
BPRS	0.071	0.049	0.775	−0.411–0.514

Abbreviations: BPRS, Brief Psychiatric Rating Scale; IFS, individual fitness score; SE, sleep efficiency; SL, sleep latency; TST, total sleep time.

* Two‐tailed *P* < 0.05.

### Longitudinal analysis of changes in IFS and sleep variables over 6 months

At T2, no significant changes in the IFS were observed over 6 months (*P* = 0.195) (Table S8). Longitudinal analysis was conducted to investigate whether changes in treatment adherence (ΔIFS) influenced changes in sleep variables (ΔSL, ΔTST, and ΔSE). Linear regression analysis was performed, using each change in sleep variable as the dependent variable, ΔIFS as the independent variable, and ΔBPRS as a covariate. No significant associations were found between ΔIFS and any of the sleep variables: ΔSL (*β* = −0.132, P = 0.535), ΔTST (*β* = 0.103, P = 0.662), or ΔSE (*β* = 0.100, P = 0.679) (Table 4). These findings suggest that under the analytical conditions of this study, no association was observed between longitudinal changes in IFS and sleep variables.

**Table 4:** Linear regression analysis adjusting for ΔBPRS: association between ΔIFS and ΔSleep variables.

Independent variable	*B*	*β*	*P*‐value	95% CI for *B* (lower–upper)
**Dependent variable: ΔSL**				
Constant	−3.901		0.062	−7.216–−0.399
ΔIFS	−0.038	−0.132	0.535	−0.146–0.068
ΔBPRS	−0.219	−0.296	0.210	−0.517–0.134
**Dependent variable: ΔTST**				
Constant	3.737		0.843	−32.543–34.900
ΔIFS	0.364	0.103	0.662	−1.481–1.969
ΔBPRS	2.221	0.247	0.255	−1.464–5.923
**Dependent variable: ΔSE**				
Constant	5.858		0.083	−0.233–10.859
ΔIFS	0.053	0.100	0.679	−0.191–0.288
ΔBPRS	0.584	0.433	0.097	−0.047–1.157

*Note*: Delta (Δ) means changes over 6 months (T2–T1).

Abbreviations: BPRS, Brief Psychiatric Rating Scale; IFS, individual fitness score; SE, sleep efficiency; SL, sleep latency; TST, total sleep time.

## DISCUSSION

This study investigated the association between clinician adherence to pharmacological guidelines for schizophrenia, measured by the IFS, and sleep variables assessed using actigraphy. These results provide meaningful insights into the relationship between guideline adherence and sleep quality in patients with schizophrenia. While a cross‐sectional analysis demonstrated a significant positive association between IFS and TST, longitudinal analyses showed no significant association between changes in IFS and changes in sleep variables over 6 months. The strengths of this study include its focus on long‐term hospitalized patients with schizophrenia, a practice more common in Japan yet rarely studied, by comparing their IFS and sleep parameters. Additionally, by selecting individuals who had been hospitalized for over 1 year, we ensured a relatively uniform living environment. This design minimizes the influence of acute symptom fluctuations and other external factors, thereby reducing bias.

### Cross‐sectional associations between IFS and sleep variables

Our study results demonstrated that greater adherence to pharmacological treatment guidelines for schizophrenia, as reflected by higher IFS scores, was associated with a longer TST at T1. This association remained significant even after adjusting for the severity of psychiatric symptoms, as measured by BPRS scores. Additionally, the data did not support the notion that groups prescribed sleep medications exhibited better sleep variables. These findings suggest that the use of sleep medications did not contribute to improved sleep outcomes in this population, which is consistent with previous studies showing that guideline‐compliant antipsychotic treatment has beneficial effects on clinical outcomes, including symptom improvement and enhanced social functioning.^21^, ^22^

The IFS was designed to assign 100 points to antipsychotic monotherapies. A 15‐point deduction was applied when one hypnotic agent was used and a 35‐point deduction was applied when two hypnotics were used. Within this scoring framework, the observed positive correlation between the IFS and TST suggests that closer alignment with antipsychotic monotherapy is associated with higher total sleep time. This finding is particularly notable. Increased wakefulness after sleep onset and reduced TST are well‐documented phenomena in untreated patients with schizophrenia. Conversely, monotherapy with second‐generation antipsychotics, such as clozapine, olanzapine, and paliperidone, has been reported to increase the TST.^12^ Although hypnotics, including benzodiazepines, orexin receptor antagonists, and melatonin receptor agonists, are known to increase TST in patients with insomnia,^28^, ^29^, ^30^ their effects in patients with schizophrenia remain unclear.

In clinical practice, the adjustment of these medications must consider the potential drug interactions, daytime somnolence, and their impact on schizophrenia symptoms. Consequently, sleep improvement should not be viewed merely as the result of individual drug effects. Nonetheless, the observed positive correlation between the IFS and TST in this study suggests that adherence to treatment guidelines may positively influence sleep duration. Specifically, prioritizing simpler prescriptions, such as monotherapy with antipsychotics, while considering the individual effects of each drug on sleep may improve sleep in patients with schizophrenia.

### Longitudinal changes in IFS and sleep variables

While cross‐sectional analysis confirmed a significant association between IFS and TST, longitudinal analysis did not reveal significant associations between changes in IFS (ΔIFS) and changes in sleep variables (ΔSL, ΔTST, and ΔSE). There are several possible explanations for these findings. The lack of statistical significance in ΔIFS during the observation period suggests that a 6‐month duration may have been insufficient to detect meaningful changes in IFS or its impact on sleep variables. The selection of a 6‐month observation period was primarily aimed at enhancing the feasibility of this study; however, future research involving similar study populations should consider extending the observation period. Although the absence of statistical significance indicates that the observed directional consistency might be coincidental, the standardized effect sizes (ΔSL, *β* = −0.132; ΔTST, *β* = 0.103; ΔSE, *β* = 0.100) all notably indicate potential improvements in sleep quality with improved IFS. Considering these findings, future studies should aim to extend the follow‐up duration and utilize adequately powered sample sizes to elucidate the impact of changes in IFS on sleep variables more clearly. Although no significant differences were found between completers and dropouts (Table S1), the supplemental longitudinal analysis—excluding dropouts—provides additional insights (Table S2). In our supplemental analysis (*n* = 33; Table S2), we observed a nominally significant reduction in SL (from 1.6 to 0.8 min, *P* = 0.042), a non‐significant yet clinically meaningful decrease in and the decrease TIB (from 527.5 to 479.0 min, *P* = 0.201), and no change in TST from 418.0 to 415.7 min, *P* = 0.416). These results suggest that guideline‐adherent pharmacotherapy may reduce daytime sedation, enhance daytime functioning, and consequently improve sleep initiation. These exploratory findings underscore the potential behavioral and physiological benefits of appropriate prescribing. Future studies should include objective assessments of daytime sleepiness and activity to clarify these mechanisms.

### Implications for clinical practice

Our study findings highlight the potential importance of adherence to pharmacological guidelines in improving sleep outcomes, specifically the TST, in patients with schizophrenia. Given the high prevalence of sleep disturbance in this population, optimizing antipsychotic prescriptions in line with guideline recommendations may represent a practical approach to addressing this critical issue.^15^ Clinicians should be encouraged to adhere to evidence‐based treatment guidelines to maximize therapeutic benefits for their patients.

Adherence to treatment guidelines, as reflected by higher IFS scores, has been linked to improvements not only in sleep but also in other domains of patient health. Longitudinal studies have shown that increases in the IFS contribute to improvements in psychotic symptoms^21^ and are positively correlated with longer working hours,^22^ suggesting that higher IFS may also benefit social functioning. Integrating the findings of this study with prior research indicates that such adherence is critical not only for sleep outcomes but also for overall clinical and functional improvements. Even in populations where guideline‐based treatment has not been fully implemented, as observed in this study, clinicians should guide patients through the process of adjusting pharmacological treatments to align with the guidelines while presenting the potential for symptom and functional improvement.

### Limitations and future directions

This study provides valuable insights into the relationship between adherence to pharmacological guidelines, as measured by the IFS, and sleep variables in patients with schizophrenia. However, this study has certain limitations. First, in this study, we applied different IFS scoring criteria for TRS and non‐TRS patients, reflecting the clinical recommendations (i.e., clozapine monotherapy was required for full IFS points in TRS). However, we did not perform subgroup analyses by diagnostic category, which is a limitation. Previous research has suggested that clozapine prescription is associated with a higher rate of antipsychotic monotherapy in TRS,^31^ potentially exerting a strong effect on IFS scoring. In our sample, no patients were prescribed clozapine at T1, while two patients were prescribed clozapine at T2. This change may have had a notable influence on the overall IFS scores and should be taken into account when interpreting the findings. Second, the findings indicated only a correlational relationship and did not establish causality. Although sleep disturbances are common in patients with schizophrenia, those without such disturbances may already have sufficient sleep, potentially leading to simpler prescriptions. Hence, these results should be interpreted cautiously. Third, the 6‐month observation period, for its feasibility in retaining participants and collecting data, may have been too short to capture meaningful longitudinal changes in IFS or sleep outcomes. Extending the observation period in future studies could help uncover the long‐term effects of guideline adherence. Fourth, the relatively small sample size posed a limitation because a post hoc analysis suggested that approximately 445 participants would be needed to detect a significant association in a longitudinal analysis. Post hoc power analysis using *R* ^2^, estimated from the standardized effect size (*β* ^2^ = 0.132^2^), indicated that a sample size of approximately 445 participants would be required to achieve 80% statistical power (*α* = 0.05). In the cross‐sectional analysis at baseline (T1), the significant IFS–TST association observed in the full cohort (*n* = 40; Tables 2 and 3) was no longer present in the completer‐only sample (*n* = 33; Tables S6 and S7), likely reflecting reduced statistical power due to the smaller sample size. Further large‐scale studies are required to validate these findings.

Fifth, at the start of this study, the participating psychiatrists had not received sufficient training in guideline‐based prescribing and had not completed the EGUIDE program,^17^, ^18^, ^19^ which likely impeded meaningful changes in IFS over the 6‐month period. Implementing educational interventions such as EGUIDE from the outset could ensure that clinicians fully understand the importance of guideline adherence.

Additionally, this study included only patients who had been hospitalized for at least 1 year to minimize confounding factors such as environmental changes and acute symptom fluctuations. Although this approach helped to stabilize the external environment, it may limit the generalizability of the findings to outpatient settings or patients with more acute presentations. Addressing these limitations in future studies by including larger and more diverse patient populations and extending the observation period may clarify the impact of guideline adherence on sleep and related clinical outcomes.

## CONCLUSION

This study underscores the importance of adherence to pharmacological guidelines in improving sleep outcomes, particularly the TST, among patients with schizophrenia. Our cross‐sectional analysis demonstrated a significant association between the IFS and TST, suggesting that guideline‐adherent prescribing behaviors can positively affect sleep. However, the absence of significant findings in the longitudinal analyses highlights the challenges of capturing dynamic changes within a limited timeframe.

Future investigations should address the limitations identified in this study by extending the observation period, increasing sample size, and incorporating a broader range of patient populations. By doing so, these studies can further validate the role of guideline adherence in improving sleep and the overall quality of life in individuals with schizophrenia. This research lays a foundation for promoting evidence‐based practices that not only improve clinical outcomes but also address broader functional and societal challenges associated with schizophrenia care.

## AUTHOR CONTRIBUTIONS

Kentaro Saito and Yusuke Arai contributed to manuscript conception. Daimei Sasayama, Toshinori Nakamura, Kazuhiro Suzuki, Mika Koido, Reiko Sahara, Yuka Nakajima, Aya Horiuchi, Fumiya Fukui, Kazuaki Kuraishi, and Shinsuke Washizuka reviewed the manuscript. Kentaro Saito and Yusuke Arai wrote the first draft of this manuscript. Daimei Sasayama, Toshinori Nakamura, and Kazuhiro Suzuki wrote the manuscript. Kentaro Saito, Yusuke Arai, Mika Koido, Reiko Sahara, Yuka Nakajima, Aya Horiuchi, Fumiya Fukui, and Kazuaki Kuraishi obtained consent, conducted interviews, and performed examinations for research. Kentaro Saito, Yusuke Arai, Daimei Sasayama, Toshinori Nakamura, and Kazuhiro Suzuki performed all statistical analyses. All authors contributed to the manuscript revision and have read and approved the submitted version.

## CONFLICT OF INTEREST STATEMENT

The authors decalre no conflicts of interest.

## ETHICS APPROVAL STATEMENT

This study protocol was approved by the Ethics Committee of Kurita Hospital (Approval No. 202302) and conducted in accordance with the Declaration of Helsinki.

## PATIENT CONSENT STATEMENT

Written informed consent was obtained from all participants.

## REGISTRY AND REGISTRATION NUMBER OF THE STUDY/TRIAL

Trial registration: A prospective observational study aimed at a comprehensive assessment of psychiatric inpatients based on objective data and prescriptions for reducing long‐term hospitalization (UMINID: 000053410), registered on January 22, 2024.

## ANIMAL STUDIES

Not applicable.

## RESEARCH INVOLVING HUMAN PARTICIPANTS

All procedures involving human participants were conducted in accordance with the ethical standards of the institutional and national research committees and the 1964 Declaration of Helsinki and its later amendments or comparable ethical standards.

## Supporting information

Supporting Information.

## Data Availability

The data that support the findings of this study are available on request from the corresponding author. The data are not publicly available due to privacy or ethical restrictions.
