# Supplementary material for: Association between pharmacological guideline adherence and actigraphy‐measured sleep variables in long‐term hospitalized patients with schizophrenia
Source: PCN Rep. 2025 Jul 10;4(3):e70154. doi: 10.1002/pcn5.70154 (PMC12241822; doi:10.1002/pcn5.70154)
Supplement: Supplementary file 1 — Supporting Information. [file PCN5-4-e70154-s001.docx]

**Supplemental materials**

Table S1. Comparing baseline characteristics (age, IFS, BPRS, and sleep variables) between individuals who dropped out at T2 and those who did not

| Characteristics | participants followed up until T2 (N = 33) | dropped out  (N = 7) | P-value ^a,b^ |
| --- | --- | --- | --- |
| Age (years) | 56.0 (51.0–61.0) | 52.0 (44.0–63.0) | .807^c^ |
| BPRS | 18.0 (6.0–23.5) | 10 .0 (2.0–21.0) | .169^c^ |
| IFS | 35.0 (0–65.0) | 10.0 (0–85.0) | .889^c^ |
| TIB (min) | 527.5 (431.0–541.3) | 519.2 (444.3–568.3) | .728^c^ |
| TST (min) | 418.0 (361.8–492.3) | 444.0 (384.5–507.3) | .651^c^ |
| SE (%) | 85.8 (77.0–92.9) | 84.6 (82.2–89.6) | .972^c^ |
| SL (min) | 1.7 (0.2–6.9) | 2.5 (0–6.3) | .676^c^ |
| ^a^The significance level is .050.  ^b^Asymptotic significance is displayed.  ^c^Exact significance is displayed for this test. | | | |

IFS: individual fitness score, BPRS: Brief Psychiatric Rating Scale, TIB: time in bed, TST: total sleep time, SE: sleep efficiency, SL: sleep latency.

Table S2. Comparison of clinical and sleep-related variables between T1 and T2 in patients who completed follow-up (n = 33).

| Characteristic | T1 (N = 33) | T2 (N = 33) | **P- value** |
| --- | --- | --- | --- |
| **Pharmacotherapy** | **Median (IQR) or N (%)** |  |  |
| Antipsychotic, CP-eq (mg) | 951.5 (607.6–1337.9) | 900.0 (600.0–1506.8) | 0.276 |
| Clozapine treatment | 0 (0%) | 2 (6.1%) | 0.492 |
| Mood stabilizer, N (%) | 11 (33.3%) | 13 (39.4%) | 0.621 |
| Benzodiazepine, N (%) | 15 (45.5%) | 14 (42.4%) | 1.000 |
| Anticholinergic, N (%) | 10 (30.3%) | 8 (24.2%) | 0.783 |
| Orexin receptor antagonist, N (%) | 13 (39.4%) | 13 (39.4%) | 1.000 |
| Melatonin receptor agonist, N (%) | 3 (9.1%) | 4 (12.1%) | 1.000 |
| Number of classes of hypnotic medications |  |  |  |
| 0, N (%) | 9 (27.3%) | 12 (36.4%) | 0.598 |
| 1, N (%) | 18 (54.5%) | 13 (39.4%) | 0.231 |
| ≥2, N (%) | 6 (18.2%) | 8 (24.2%) | 0.566 |
| IFS | 35.0 (0–65.0) | 40.0 (0–85.0) | 0.195 |
| **Symptom rating scales** | **Median (IQR)** |  |  |
| BPRS | 18.0 (6.0–23.5) | 9.0 (4.5–13.0) | 0.002 |
| **Sleep variables based on actigraphy** | **Median (IQR)** |  |  |
| TIB (min) | 527.5 (431.0–541.3) | 479.0 (451.5–525.8) | 0.201 |
| TST (min) | 418.0 (361.8–492.3) | 415.7 (365.0–467.9) | 0.416 |
| SE = TST/TIB (%) | 85.8 (77.0–92.9) | 87.6 (79.7–92.2) | 0.526 |
| SL (min) | 1.6 (0.2–6.9) | 0.8 (0–4.6) | 0.042 |

IFS: individual fitness score, BPRS: Brief Psychiatric Rating Scale, TIB: time in bed, TST: total sleep time, SE: sleep efficiency, SL: sleep latency.

Table S3. Differences in SL among groups divided by the number of classes of hypnotic medications

| Differences in SL | | | | | |
| --- | --- | --- | --- | --- | --- |
| Number of classes | Test Statistic | Std. Error | Std. Test Statistic | P-value | Adj. P-value ^a^ |
| ≥2 vs. 1 | 8.350 | 4.851 | 1.721 | .085 | .256 |
| ≥2 vs. 0 | 9.000 | 5.293 | 1.700 | .089 | .267 |
| 1 vs. 0 | .650 | 4.235 | .153 | .878 | 1.000 |
| Each row tests the null hypothesis that the Sample 1 and Sample 2 distributions are the same.  Asymptotic significances (2-sided tests) are displayed. The significance level is .050. | | | | | |
| ^a^Significance values have been adjusted using Bonferroni correction for multiple tests. | | | | | |

SL: sleep latency

Table S4. Differences in TST among groups divided by the number of classes of hypnotic medications

| Differences in TST | | | | | |
| --- | --- | --- | --- | --- | --- |
| Number of classes | Test Statistic | Std. Error | Std. Test Statistic | P-value | Adj. P-value ^a^ |
| ≥2 vs. 1 | 1.700 | 4.890 | .348 | .728 | 1.000 |
| ≥2 vs. 0 | 6.333 | 5.336 | 1.187 | .235 | .706 |
| 1 vs. 0 | 4.633 | 4.269 | 1.085 | .278 | .833 |
| Each row tests the null hypothesis that the Sample 1 and Sample 2 distributions are the same.  Asymptotic significances (2-sided tests) are displayed. The significance level is .050. | | | | | |
| ^a^Significance values have been adjusted using Bonferroni correction for multiple tests. | | | | | |

TST: total sleep time

Table S5. Differences in SE among groups divided by the number of classes of hypnotic medications

| Differences in SE | | | | | |
| --- | --- | --- | --- | --- | --- |
| Number of classes | Test Statistic | Std. Error | Std. Test Statistic | P-value | Adj. P-value ^a^ |
| 1 vs. 0 | 1.133 | 4.269 | .265 | .791 | 1.000 |
| 1 vs. ≥2 | -2.300 | 4.890 | -.470 | .638 | 1.000 |
| 0 vs. ≥2 | -1.167 | 5.336 | -.219 | .827 | 1.000 |
| Each row tests the null hypothesis that the Sample 1 and Sample 2 distributions are the same.  Asymptotic significances (2-sided tests) are displayed. The significance level is .050. | | | | | |
| ^a^Significance values have been adjusted using Bonferroni correction for multiple tests. | | | | | |

Table S6. Correlation Between IFS and Sleep Variables in patients who completed follow-up (n = 33).

| Variable | rho | P-value | 95% CI (Lower- Upper) |
| --- | --- | --- | --- |
| IFS-TST | 0.287 | 0.105 | -0.072–0.581 |
| IFS-SL | -0.153 | 0.395 | -0.480–0.211 |
| IFS-SE | 0.194 | 0.278 | -0.170–0.512 |

Abbreviations: IFS: individual fitness score, TST: total sleep time, SL: sleep latency, SE: sleep efficiency

Table S7. Linear Regression Analysis Adjusting for BPRS: Association Between IFS and Sleep Variables in patients who completed follow-up (n = 33).

| **Dependent variable: SL** |  |  |  |  |
| --- | --- | --- | --- | --- |
| Independent variable | B | β | P-value | 95% CI for B (Lower-Upper) |
| (Constant) | 12.297 |  | 0.011 | 3.055–21.540 |
| IFS | -0.082 | -0.289 | 0.136 | -0.192–0.027 |
| BPRS | -0.199 | -0.195 | 0.311 | -0.593–0.195 |
| **Dependent variable: TST** |  |  |  |  |
| Independent variable | B | β | P-value | 95% CI for B (Lower-Upper) |
| (Constant) | 407.588 |  | <0.001 | 305.502–509.675 |
| IFS | 0.690 | 0.220 | 0.254 | -0.521–1.900 |
| BPRS | -1.205 | -0.107 | 0.576 | -5.558–3.147 |
| **Dependent variable: SE** |  |  |  |  |
| Independent variable | B | β | P-value | 95% CI for B (Lower-Upper) |
| (Constant) | 81.717 |  | <0.001 | 67.098–96.335 |
| IFS | 0.045 | 0.103 | 0.602 | -0.129–0.218 |
| BPRS | -0.099 | -0.063 | 0.747 | -0.723–0.524 |

Abbreviations: IFS: individual fitness score, BPRS: Brief Psychiatric Rating Scale, SL: sleep latency, TST: total sleep time, SE: sleep efficiency

Table S8. Differences in IFS values over 6 months employing related-sample Wilcoxon signed-rank test

|  | all participants  (N = 40) | participants followed up until T2 (N = 33) | P-value ^a,b^ |
| --- | --- | --- | --- |
| IFS | 35.0 (0–65.0) | 40.0 (0–85.0) | .195 |
| ^a^The significance level is .050.  ^b^Asymptotic significance is displayed. | | | |

IFS: individual fitness score
